# Evolution of BACON Domain Tandem Repeats in crAssphage and Novel Gut Bacteriophage Lineages

**DOI:** 10.3390/v11121085

**Published:** 2019-11-21

**Authors:** Patrick A. de Jonge, F. A. Bastiaan von Meijenfeldt, Laura E. van Rooijen, Stan J. J. Brouns, Bas E. Dutilh

**Affiliations:** 1 Theoretical Biology and Bioinformatics, Science4 Life, Utrecht University, 3584 CH Utrecht, The Netherlands; p.a.dejonge@uu.nl (P.A.d.J.); bastiaanvonmeijenfeldt@gmail.com (F.A.B.v.M.); lauraelsevanrooijen@gmail.com (L.E.v.R.); 2 Department of Bionanoscience, Kavli Institute of Nanoscience, Delft University of Technology, 2629 HZ Delft, The Netherlands; s.j.j.brouns@tudelft.nl; 3 Centre for Molecular and Biomolecular Informatics, Radboud Institute for Molecular Life Sciences, Radboud University Medical Centre, 6525 GA Nijmegen, The Netherlands

**Keywords:** Bacteroides-associated carbohydrate-binding often N-terminal domain, BACON domain, protein domain tandem arrays, bacteriophage, gut virome, crAssphage, metagenomics, phage tail-associated protein domains, genome evolution

## Abstract

The human gut contains an expanse of largely unstudied bacteriophages. Among the most common are crAss-like phages, which were predicted to infect *Bacteriodetes* hosts. CrAssphage, the first crAss-like phage to be discovered, contains a protein encoding a *Bacteroides*-associated carbohydrate-binding often N-terminal (BACON) domain tandem repeat. Because protein domain tandem repeats are often hotspots of evolution, BACON domains may provide insight into the evolution of crAss-like phages. Here, we studied the biodiversity and evolution of BACON domains in bacteriophages by analysing over 2 million viral contigs. We found a high biodiversity of BACON in seven gut phage lineages, including five known crAss-like phage lineages and two novel gut phage lineages that are distantly related to crAss-like phages. In three BACON-containing phage lineages, we found that BACON domain tandem repeats were associated with phage tail proteins, suggestive of a possible role of these repeats in host binding. In contrast, individual BACON domains that did not occur in tandem were not found in the proximity of tail proteins. In two lineages, tail-associated BACON domain tandem repeats evolved largely through horizontal transfer of separate domains. In the third lineage that includes the prototypical crAssphage, the tandem repeats arose from several sequential domain duplications, resulting in a characteristic tandem array that is distinct from bacterial BACON domains. We conclude that phage tail-associated BACON domain tandem repeats have evolved in at least two independent cases in gut bacteriophages, including in the widespread gut phage crAssphage.

## 1. Introduction

Bacteriophage populations are essential for stability and proper functioning of the human gut microbiome [1]. Gut phages may provide immunity against bacterial pathogens [2], and changes in gut virome composition have been observed in diseases such as inflammatory bowel disease [3], type I diabetes [4], malnutrition [5] and colorectal cancer [6]. Although the role of phage populations in the gut microbiome is increasingly better understood, the role of individual phage species remains almost completely unknown. This last fact is exemplified by crAssphage, a phage that was first described from metagenomic datasets in 2014. Since then, crAssphage has been recognised as the prototypical member of an expansive family of phages [7] that is abundant in the human gut [8]. The crAss-like phages are distributed globally [9], present in approximately half the human population [9,10], and very abundant in some individual gut viromes [11]. However, little is known about the role, evolution and distinguishing characteristics of specific crAss-like phage lineages.

One characteristic crAssphage protein (gp64 in NC_024711.1) encodes a tandem repeat of eight *Bacteroides*-associated carbohydrate-binding often N-terminal (BACON) domains [10]. BACON domains are most commonly found in carbohydrate-binding proteins of *Bacteroidetes* bacteria, but despite their name, a direct role in carbohydrate-binding for BACON domains is yet to be proven. The only currently published BACON domain X-ray crystallography structure in human gut *Bacteroidetes* indicated that they form links between carbohydrate-binding domains and are anchored onto the surface of *Bacteroides* cells and do not perform a carbohydrate-binding function themselves [12]. This X-ray crystallography structure also indicated that BACON domains form fibrous immunoglobulin-like (ig-like) β-sandwiches [12]. This matches the association between BACON domains and carbohydrate-binding proteins, as bacterial and viral ig-like domains are often associated with proteins possessing binding functions [13]. Phage ig-like domains are implicated in binding to phage hosts [14] and carbohydrates [15], and are common in surface proteins such as tail fibres. In the tail fibres and spikes of taxonomically diverse phages like T4 [16,17], p2 [18], HK620 [19], p22 [20,21] and Sf6 [22], ig-like and other β-hairpin folded domains commonly form tandem repeats [23]. Likewise, in crAssphage the ig-like BACON domains form a tandem repeat.

Proteins with domain tandem repeats are found throughout the tree of life [24]. Because expansions of domain tandem repeats are a marker for adaptation to new environments, these structures are considered to be evolutionary hotspots [25]. Repeat expansions often involve duplications of multiple domains [26] that occur internally in the tandem array and not at the protein termini, as is common in non-tandem repeated domains [26]. Following tandem domain duplications, the independent units in a repeat rapidly evolve until only residues necessary for correct folding are conserved [27]. Once established in this manner, and contrasting their status as evolutionary hotspots, domain tandem repeats are often stable over long evolutionarily timespans [28]. As a result of their evolutionary mechanism, domain tandem repeats can be used to study the evolution and adaptational history of species. This is particularly true in eukaryotes, where they are well studied [24]. In the context of rapid and reticulate phage evolution, much less is known about domain tandem repeat evolution.

Here we studied the evolution of BACON domain tandem repeats in crAss-like phages. First, we constructed a profile hidden Markov model (HMM) of the crAssphage-like BACON domain (crAss-BACON), which we identified in half of the known candidate crAss-like phage lineages and in two novel gut phage lineages. Tandem repeats of the crAss-BACON were exclusive to two crAss-like phage candidate genera and one of the novel lineages, and were always genomically flanked by tail proteins. In phage lineages with single (non-repeated) crAss-BACONs, they were associated with the replicative and head regions of the phage genome. By studying crAss-BACON evolution, we showed that crAss-BACON tandem repeats are divergent from bacterial domains, whereas individually occurring crAss-BACON domains are more closely related to bacterial sequences, indicating frequent horizontal transfer between viral and cellular organisms. Finally, we showed that the crAss-BACON tandem repeat in the most widespread crAss-like phage lineage has evolved through multiple single-domain duplications.

## 2. Materials and Methods

### 2.1. Data

We identified BACON domains across a broad range of viruses using two datasets that included both genomic and metagenomic sequences. The first dataset consisted of 661 crAss-like phage sequences from five recent publications [7,9,10,11,29] that ranged in size from short fragments of 623 bp to complete and near-complete genomes of up to 104,752 bp (median: 27,648 bp). The second dataset was more diverse and consisted of 2,147,193 viral contigs from four recent viromics papers [30,31,32,33]. The contigs in the second dataset originated from ecosystems across the biosphere, including host-associated microbiomes from humans and other animals, fresh and saltwater aquatic ecosystems, and marine and terrestrial sediments (see Table S1, Supplementary Materials, for metadata on contigs in which crAss-BACONs were identified). A profile hidden Markov model (HMM) of the BACON domain (PF13004), constructed from 304 protein domains present in *Bacteroidetes* bacteria, was downloaded from the Pfam database v32.0 on 1 April 2019 [34]. The 304 bacterial BACON domain sequences, from which PF13004 was constructed, were also retrieved from Pfam.

### 2.2. Identification of BACON Domains

All bioinformatics tools mentioned below were run with default settings and cut-offs, except where stated explicitly.

Before searching for BACON domains, we predicted all ORFs in both datasets of viral sequences using Prodigal v2.6.1 [35] with translation table 11. BACON domains were identified in the dataset of crAss-like phage sequences using iterative searches with hmmsearch v3.1b2 [36]. In the first iteration, PF13004 was used as profile HMM for the search against the crAss-like phage dataset. For subsequent iterations, profile HMMs were constructed using hmmbuild v3.1b2 with the aligned domain hits of the previous iteration as input. A total of four iterations were performed, at which point the number of hits converged. The final dataset of crAss-BACONs contained 605 domains in 171 ORFs on 122 contigs. A crAss-BACON profile HMM was made with these domains as above, which was subsequently used to search in the metagenome dataset with hmmsearch v3.1b2 [36]. This search identified an additional 801 BACON domains in 296 ORFs on 246 contigs.

### 2.3. Clustering of BACON Domain-Containing Viral Contigs

To obtain a tentative taxonomical classification of the crAss-BACON-containing contigs, we clustered them based on shared protein content [37,38,39]. To increase clustering reliability, only contigs with more than 10 ORFs were retained. This decreased the dataset to 1205 BACON domains in 376 ORFs on 286 contigs. This dataset was used for all subsequent analyses. For a full overview of all domains, ORFs and contigs in this dataset and related metadata, see Tables S1 and S2, Supplementary Materials. Homologous gene clusters were made by performing a BLASTp all versus all on every ORF from every crAss-BACON-containing contig using Diamond v0.9.25 [40]. The output was filtered for hits with a bit score >50. Subsequently, homologous ORF clusters were formed using the Markov cluster (MCL) algorithm with inflation parameter 2 (option -I 2) [41]. The number of shared homologous gene clusters between each contig pair was determined, from which the significantly shared gene content was calculated using the R function phyper. A Euclidean distance matrix of the hypergeometric *p*-values was constructed with the R function dist and used for clustering with the Ward.D2 algorithm as incorporated in R. The optimal number of clusters was determined using the NbClust v3.0 R package [42], whereas silhouette plots for the clusters were obtained using the factoextra v1.0.5 R package. Heatmaps were plotted using the heatmaply v0.16.0 R package [43].

To analyse the conservation of the cluster 1 crAss-BACON tandem repeat, all cluster 1 crAss-BACON ORFs with eight domains were selected and aligned with Clustal Omega v1.2.1 [44]. Information content and residue conservation scores per position of the alignment were calculated using Geneious v.9.1.8 [45].

### 2.4. Analysis of Novel Contig Clusters

Most crAss-BACON-containing contig clusters contained members of previously defined candidate crAss-like phage subfamilies and genera, except clusters A and B which consisted of highly dissimilar viral sequences (see Results and Discussion). To provide an initial sequence-based characterization of these novel viruses, the largest contigs from each cluster were extracted and annotated using the PROKKA v1.11 software tool [46], with the metagenomics option enabled in two separate runs for both the viral and bacterial settings (i.e., with --metagenome and both --kingdom Bacteria and --kingdom Viruses options). Whole genome comparisons between the largest contig of each cluster were made using Easyfig v2.2.3 [47], which performs tBLASTx searches along the entirety of two sequences. Easyfig employed the tBLASTx function of BLAST+ v2.9.0 [48].

To further study the relation of the newly described contig clusters to known crAss-like phages, we performed a phylogenetic analysis of crAss-like phage head proteins. Five crAss-like phage head proteins that were previously used to determine crAss-like phage taxonomy [7] were extracted from the crAssphage genome (locus tags gp73–77 in NC_024711.1). They were used as queries for jackhmmer searches [36] against a database consisting of all predicted ORFs from the 166 crAss-BACON-containing contigs longer than 85 kbp. Analysis of the jackhmmer output showed that two of the five head proteins (gp76 and 77) had homologs in all but one of the phage clusters. These two proteins were selected for phylogenetic analysis. A separate alignment was made for each head protein using Clustal Omega v1.2.1 [44]. The alignments of the head proteins were concatenated and positions with more than 95% gaps were removed using trimal v1.2 [49] (option -gt 0.05). As was recommended in a recent meta-analysis of phylogenetic tree reconstruction tools [50], ten maximum likelihood trees were made using IQ-Tree v1.6 [51] using model finder [52] and 1000 iterations of both the SH-like approximate likelihood ratio test and the ultrafast bootstrap approximation (UFBoot) [53] (i.e., options -alrt 1000 -bb 1000). Out of the ten constructed trees, the one with the highest likelihood (for which model finder selected VT+F+R8) was visualised using interactive Tree of Life v4.4.2 [54].

Hosts were predicted for all crAss-BACON contigs with a length over 75,000 bp using the host prediction algorithm WiSH v1.0 [55]. This program uses k-mer profiles of bacteria and phages to calculate likelihood scores for a given phage–host interaction, with the highest log-likelihood score denoting the most likely host. As bacterial genomes, we used 2613 complete bacterial genomes that were extracted from the PATRIC database that was downloaded on 20 June 2019 [56]. A single genome sequence was selected for every genus in the database, with the highest score according to the formula C – 5 * M, where C and M are the CheckM completeness and contamination scores, respectively [57]. In case of a draw, the genome with the highest coarse consistency score was selected. These values were provided by the PATRIC database.

### 2.5. Examining of BACON ORF Genetic Neighbourhoods

To study the genomic neighbourhood of crAss-BACON ORFs, these plus five ORFs up- and downstream were collected. All ORFs were queried against the NCBI non-redundant protein sequences collection [58] with BLASTp on the BLAST webserver [59] on 26 June 2019. In some contigs, the neighbourhood search only returned significant similarity (*E*-value ≤10^−5^) to proteins with a “hypothetical protein” function description. Because this provided no additional insight into the role of these 121 crAss-BACON ORFs, they were discarded from the analysis. All other neighbourhoods were plotted using the ggplot2 v3.1.0 R package.

### 2.6. Phylogenetic Analysis of BACON Domains

To study crAss-BACON evolution, we constructed an approximate maximum likelihood tree. CrAss-BACONs were first aligned with Clustal Omega v1.2.1 [44]. While crAss-BACONs are homologous, they possess relatively low sequence similarity, as is characteristic for tandem domain repeats [27]. To improve likelihood and bootstrapping support of the phylogenetic analysis, we trimmed positions with more than 60% gaps using trimal v1.2 [49] (option -gt 0.4). The resulting alignment maintained all four highly conserved crAss-BACON residues (see Results and Discussion). The tree was subsequently constructed as described above in Section 2.4. Out of the ten constructed trees, the one with the highest likelihood (for which model finder selected WAG+R5) was visualised using interactive Tree of Life v4.4.2 [54].

To analyse whether the cluster 1 crAss-BACON array had evolved through simultaneous duplication of multiple domains, the eight domains from crAssphage were subjected to a BLASTp all versus all using BLAST+ v2.9.0 [48], and bitscores were plotted, as has been described previously [26].

## 3. Results and Discussion

### 3.1. Construction of a Specific Profile HMM of the crAss-Like Phage BACON Domain

**Figure 1:**
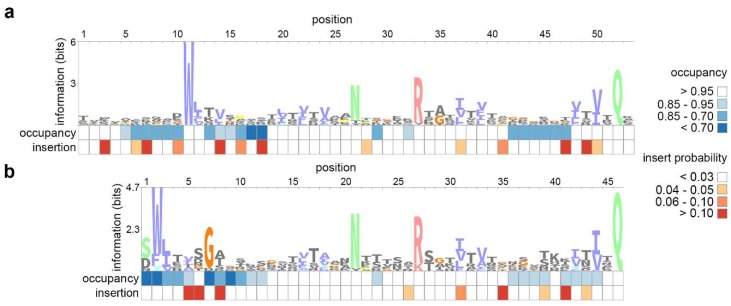
Sequence logos of *Bacteroides*-associated carbohydrate-binding often N-terminal (BACON) domain profile hidden Markov models (HMMs) show the divergence between (**a**) the bacterial BACON domain and (**b**) the crAssphage-like BACON domain (crAss-BACON). Profile HMMs were constructed from (**a**) 304 *Bacteroidetes* domains (PF13004) and (**b**) domains identified in crAss-like phages. These sequence logos are representations of profile HMMs, which contain probability scores for each amino acid residue at each position in an alignment. In addition, profile HMMs contain probability statistics for insertions and deletions at each position. Occupancy scores denote the probability that an amino acid residue is found at a given position (i.e., low values mean a deletion is more likely to occur at that position). Insertion scores denote the probability of an insertion after a given location. Images were constructed with the Skylign webserver [61,62].

We started our study of BACON domain diversity and evolution by constructing a profile HMM distinctive to crAssphage-like BACON domains (crAss-BACONs). The *Bacteroides*-derived BACON domain that was available in Pfam (PF13004) [34] contained 4 highly conserved residues [26,27] out of a total of 45 (Figure 1a) [60]. These are an N-terminal tryptophan (position 11 in Figure 1a), a central asparagine and arginine (positions 27 and 33) and a C-terminal glutamine (position 52). To improve identification of crAss-BACONs with few conserved residues, we performed iterated distant homology searches (see Materials and Methods). This iterative search identified 605 BACON domains in 122 crAss-like contigs after four iterations. With these 605 domains, a crAss-BACON profile HMM was made. Comparison between the crAss-BACON profile HMM and PF13004 showed conservation of all four characteristic BACON residues (Figure 1a,b). The main difference between PF13004 and crAss-BACON profile HMM was a shorter N-terminus of the domain. The first 10 residues of the domain were absent altogether, while low occupancy scores in the profile HMM show that some of the 10 subsequent residues were absent from up to 30% of crAss-BACONs. High insert probabilities within the first 10 residues of both profile HMMs further show that this region of the domain is flexible.

The dataset in which we searched for BACON domains included contigs from an earlier study that presented a preliminary crAss-like phage taxonomy [11]. That study classified human gut-associated crAss-like phages into ten candidate genera and four candidate subfamilies. We identified crAss-BACONs in all four candidate subfamilies including *Alpha-*, *Beta-*, *Gamma-* and *Deltacrassvirinae*, but not in all ten candidate genera. Candidate genera I, II, III, V, VI and VII contained crAss-BACONs, whereas IV, VIII, IX and X did not. *Alphacrassvirinae* is the most widespread subfamily that contains candidate genus I and the prototypical crAssphage. This subfamily contains two candidate genera with crAss-BACONs (I and III) and two without it (IV and IX). BACON domains are particularly prevalent in candidate genus I, where 55 of the 63 genomes that were previously categorised in this genus contain crAss-BACONs. In candidate genus III, 7 of the 22 genomes contain crAss-BACONs. Likewise, *Deltacrassvirinae* contains one candidate genus with crAss-BACONs (VII, crAss-BACONs found in 10 of the 37 genomes) and two without it (VIII and X). We found crAss-BACONs in *Betacrassvirinae* (candidate genus VI, crAss-BACONs in 3 of the 22 genomes), which contains the first isolated crAss-like phage [63], and in *Gammacrassvirinae* (II and V, crAss-BACONs in 13 of 14, and 15 of 18 genomes, respectively). Thus, crAss-BACONs were absent from some lineages, even though the search space contained genome sequences from all established candidate crAss-like phage genera that a previous publication showed were (near-)complete [11]. We conclude that crAss-BACONs are widespread but not universally conserved among the taxonomically diverse crAss-like phage lineages.

### 3.2. BACON Domains Are Found in Diverse Phages

As BACON domains were found in taxonomically diverse crAss-like phages, we next determined how widespread they were in other phages. For this, we queried 2,147,193 viral sequences from seven metaviromics studies for the crAss-BACON profile HMM (Figure 1b) and identified 801 BACON domains in 246 contigs. Despite using a search space with viral contigs from a wide variety of ecosystems, contigs where crAss-BACONs were identified largely originated from human intestinal or sewage datasets. Using a profile HMM for sequence similarity searches allows for detecting considerably divergent sequences. Still, the crAss-BACON profile HMM was initially constructed from crAss-like phage sequences, so it is possible that very divergent BACON domains may have been missed. Additional biomes where crAss-BACON containing contigs were found include cow rumen (*n* = 2), chicken ceca (*n* = 2), sheep rumen (*n* = 1) and wombat intestines (*n* = 1), as well as three contigs originating from human oral metagenomes [30] (see Table S1, Supplementary Materials).

**Figure 2:**
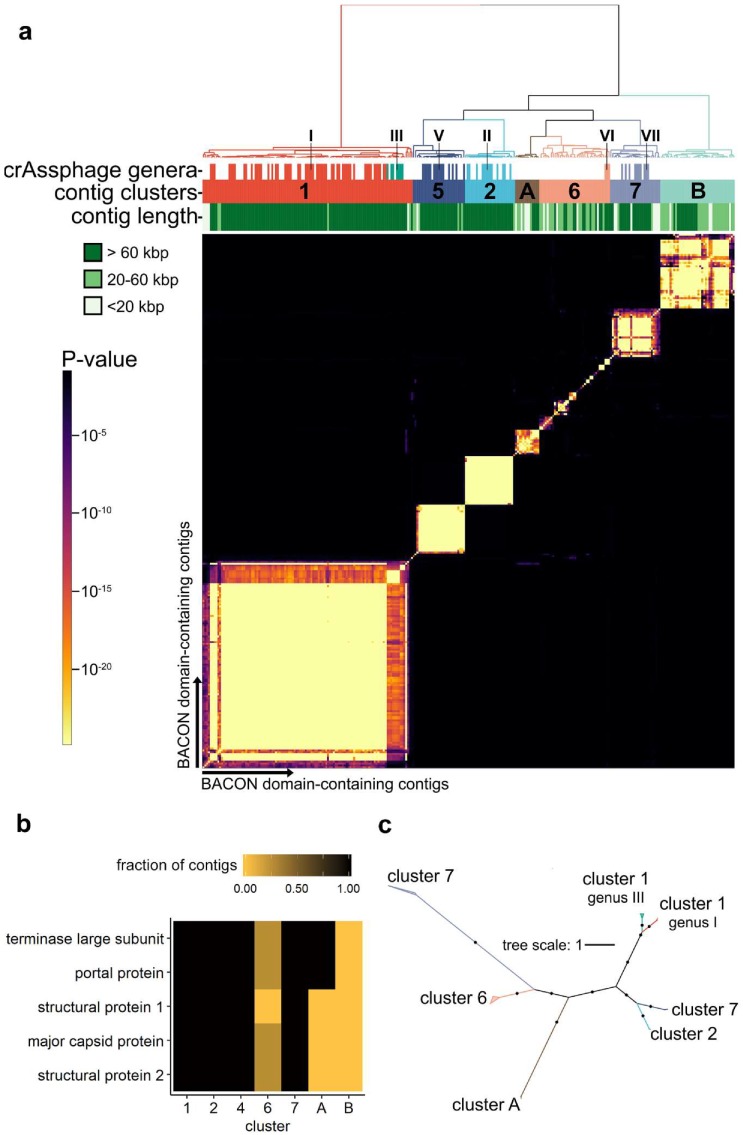
crAss-BACON-containing contigs form seven distinct clusters, some of which are distantly related to crAss-like phages. (**a**) Heatmap of crAss-BACON-containing phage contigs with seven clusters denoted. Clustering was performed based on a hypergeometric *p*-value calculated from the number of shared homologous ORFs between each pair of contigs (see Materials and Methods). Marks in the crAssphage genera row indicate contigs which have previously been identified as members of one of the ten candidate crAss-like genera. Roman numerals indicate the candidate crAss-like genera to which contigs belong [11]. (**b**) Variable presence of conserved crAssphage-like head proteins among the seven clusters. The fraction denotes the fraction of contigs in the cluster that contained that protein. No homologs were found from 1/5 proteins in cluster 6 contigs, 3/5 in cluster A and 5/5 in cluster B. (**c**) Approximate maximum likelihood tree of crAss-like terminase and portal protein homologs. Black dots indicate branches with ultrafast bootstrap support >90. Names of crAss-like phage candidate genera are according to Guerin et al. [11]. No clade with cluster B contigs is present due to the absence of crAss-like terminase and portal proteins from the contigs of this cluster.

To investigate the biodiversity of the organisms containing crAss-BACONs, we quantified the similarity of these contigs through their shared protein family content [37,38,39]. Hierarchical clustering of significantly shared protein family content per contig pair resulted in seven distinct clusters (Figure 2a). Five clusters contained contigs that were previously proposed as candidate crAss-like phage genera [11]. Cluster 1 represents the *Alphacrassvirinae* candidate subfamily and contains contigs from candidate genera I and III. Clusters 2, 5 and 7 conform with candidate genera II, V and VII, respectively. As previously observed for candidate genus VI [11], cluster 6 is highly diverse (see also Figure S1, Supplementary Materials). The remaining two clusters, which we labelled A and B, contain contigs that were not previously identified as crAss-like phages.

We next determined how the seven clusters were related by performing a phylogenetic analysis of five crAss-like phage head proteins that are conserved in all crAss-like phages [7]. To maximize correct phylogenetic inference, we selected only contigs over 85 kbp for this analysis. The five crAss-like phage head proteins included a terminase and a major capsid protein, often among the most conserved phage proteins [64,65]. Conversely to this and the conservation of these proteins in known crAss-like phages, one of the head proteins was absent from all cluster 6 contigs, whereas the other four were present in a quarter of the contigs in this cluster (Figure 2b). Their absence likely suggests that cluster 6 includes multiple phage lineages and/or incomplete genome fragments. The five head proteins were also partially or completely absent from clusters A and B. Cluster A contigs contained only the terminase and portal proteins, while cluster B contained no homologs to any of the five head proteins (Figure 2b). This suggests that cluster A is more closely related to crAss-like phages than cluster B. A phylogenetic analysis of the concatenated multiple sequence alignment of the two head proteins, which had homologs in all clusters except B, revealed that clusters 7 and A were the most distant lineages (Figure 2c). Considering that all five head proteins are present in cluster 7 but not in cluster A, we suggest that cluster 7 is more closely related to the other crAss-like phages than cluster A.

To further analyse the phage lineages in clusters A and B, we functionally annotated the protein-encoding genes on the longest sequences from clusters A and B. These contigs were 154,123 bp (cluster A) and 87,116 bp (cluster B) long and contained 180 and 110 ORFs respectively (see Tables S3 and S4, Supplementary Materials for annotation tables). Of the 180 ORFs in the cluster A representative contig, 96 had significant hits to the nr database. Most of these hits (64 hits) were found in *Bacteroidetes* species. Ten of the first fifteen genes on this contig hit genes in a *Parabacteroides merdae* genomic region. As the seventeenth gene on the *P. merdae* contig is a transposase, this region likely signifies a partial prophage [66]. This assertion is strengthened by the presence of two phage antirepressor proteins, which act in prophage induction [67]. About half the ORFs (56 out of 110) in the representative cluster B contig hit *Proteobacteria* proteins, specifically from *Acinetobacter baumannii* (35 ORFs) and *Klebsiella pneumoniae* (14 ORFs). Multiple cluster B ORFs hit phage structural proteins located in bacterial genomes, including three in *A. baumannii,* one in *K. pneumoniae*, one in *Desulfovibrio alaskensis* and one in *Rhodobacteriacea.* Like the cluster A contig, the cluster B contig may thus have a temperate lifestyle. The high number of ORFs with hits in *Proteobacteria*, coupled with only three ORF hits to *Bacteroidetes* species, could indicate that this cluster B phage infects *Proteobacteria*, a different phylum than the crAss-like phages [7,63]. If this were to be confirmed, it would mean that BACON domains are not as exclusive to the *Bacteroidetes* as previously thought [60]. In contrast, the WIsH host-prediction algorithm (published accuracy of 60%) did predict *Bacteroidetes* hosts for cluster B contigs, consistent with crAss-like phages [55]. These conflicting results highlight the importance of experimental research to make firm conclusions about phage host range [68,69].

Few ORFs in either the cluster A or B contigs are homologous to phage proteins in the RefSeq database (see Tables S3 and S4, Supplementary Materials). Of the two, cluster A had the most hits to known phages. These included 12 ORFs with homology to *Cellulophaga* phages phi17:2 and phi4:1 proteins, which represented roughly 10% of the ORFs predicted on the cluster A contig. Since *Cellulophaga* phage phi14:2 is a distant relative of crAssphage [7], this is a further indication that cluster A is distantly related to crAss-like phages. Only five cluster B ORFs showed direct similarity to proteins from isolated phages (BLASTp *E*-value <10^−5^). These phages infect four different phyla and are unrelated to crAss-like phages. The above results show that cluster A and B genomes likely represent newly described phage lineages.

### 3.3. BACON Domains Have Diverse Configurations in Phages

**Figure 3:**
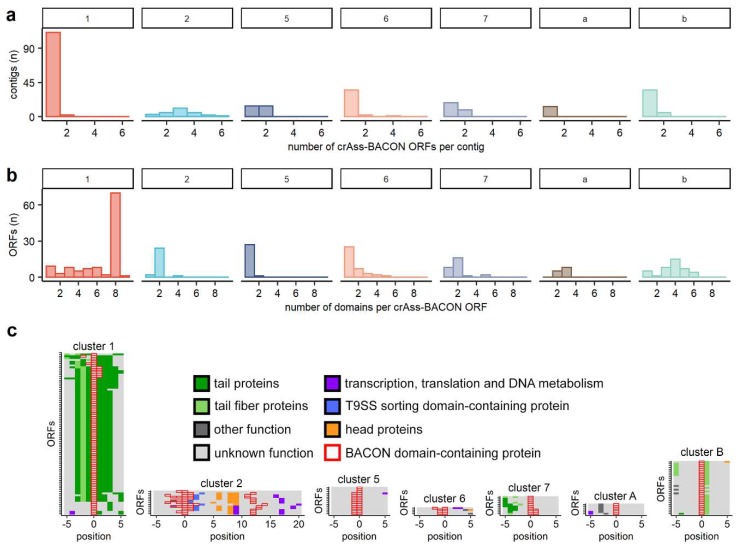
BACON-containing ORFs have diverse domain architectures and genomic neighbourhoods. Histograms of (**a**) the number of BACON domains per ORF and (**b**) the number of BACON domains containing ORFs per contig show the diversity in domain architecture between clusters. (**c**) The genomic neighbourhood of crAss-BACON ORFs. Note that the ORFs containing crAss-BACON tandem arrays (clusters 1,7 and B) are flanked by tail proteins. Only contigs with more than 10 ORF predictions and at least one homolog with a predicted function other than “hypothetical protein” are shown (cluster 1: *n* = 88/113 contigs are shown, 2: *n* = 13/27, 5 = 15/28, 6 = 4/38, 7 = 10/27, A = 6/13, B = 29/40).

After establishing that crAss-BACONs are widespread in crAss-like phages and beyond, we next looked at differences in crAss-BACON architecture among the phage clusters. Domain tandem repeats in cellular organisms tend to rapidly evolve following domain duplications, after which they remain stable and conserved within species [28]. The tandem repeat of eight crAss-BACONs per ORF, as found in the prototypical crAssphage [10], was unique to cluster 1 (Figure 3a), where the crAss-BACON sequences were highly conserved (Figure S2, Supplementary Materials).

Other crAss-BACON architectures were found outside of cluster 1 where all crAss-BACON-containing ORFs had fewer than eight domains. Most crAss-BACON ORFs in clusters 2, 5 and 6 contain a single domain (Figure 3a). Multiple contigs in clusters 2 and 5 possessed multiple crAss-BACON ORFs (Figure 3b), which in some cases were separated by several other ORFs (Figure 3c). Like cluster 1, most crAss-BACON ORFs in clusters 7, A and B contained more than one domain (Figure 3a). While crAss-BACONs in cluster A ORFs were not organized in a tandem array, clusters 7 and B contained instances of domain tandem repeats of five and six domains. Therefore, crAss-BACON tandem repeats are limited to clusters 1, 7 and B.

Because repeats of β-hairpin folds such as BACON are common in phage tails [23], we hypothesised that the crAss-BACON ORFs in cluster 1, 7 and B may encode tail-associated proteins. The poor conservation of the C-terminus of cluster 1 crAss-BACON ORFs (Figure S2) might be consistent with their role as tail fibres, as phage receptor-binding proteins commonly have low conservation in one terminus [70]. We could not directly verify the function of crAss-BACON tandem repeat-containing ORFs, as only two cluster 2 crAss-BACON ORFs had sequence similarity to proteins from the nr database with functional predictions. These two hits, to a putative glycosyl hydrolase (WP_116811532.1, *E*-value of 6 × 10^−8^) and a putative T9SS sorting domain-containing protein (WP_091894873.1, *E*-value of 9 × 10^−8^), were solely based on an alignment to BACON domains. To circumvent this lack of detectable sequence similarity of crAss-BACON ORFs to proteins with predicted function, we instead analysed their genomic neighbourhoods (Figure 3c). This showed that ORFs with crAss-BACON tandem repeats in clusters 1, 7 and B are located near predicted tail or tail fibre proteins (Figure 3c). The crAss-BACON ORFs from cluster 1 in particular are flanked by multiple predicted genes encoding phage tail proteins. In clusters 2, 5, 6 and A, the crAss-BACON-containing ORFs are in proximity of proteins with a variety of other predicted functions. In cluster 2, we identified multiple crAss-BACON ORFs that were located on either side of the head section of the genome. Upstream of the head section were crAss-BACON ORFs and T9SS sorting domain-containing proteins. In one case, a crAss-BACON ORF also contained a T9SS sorting domain. The T9SS secretion system, which is only found in *Bacteroidetes*, is used for transport of virulence factors, nutrient acquisition and gliding motility [71]. How this relates to the presence of BACON domains and their correlation to carbohydrate-binding requires additional research.

**Figure 4:**
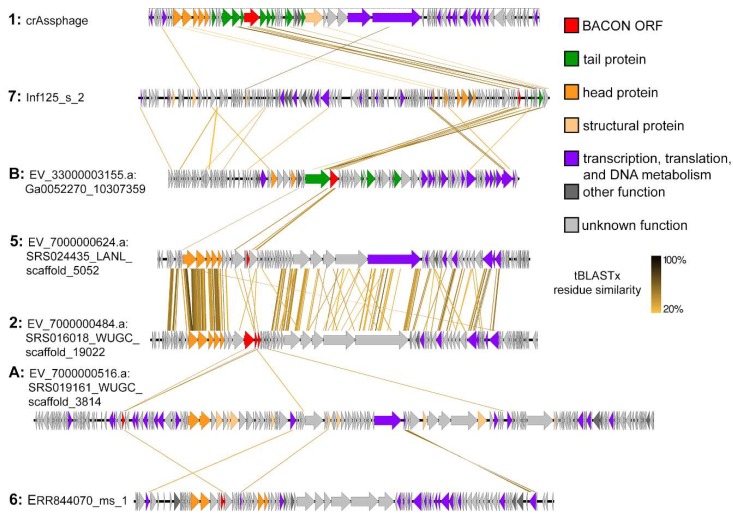
Whole genome comparisons of selected contigs from each phage cluster show that horizontal transfer has occurred in the neighbourhood of crAss-BACON ORFs between the otherwise unsimilar clusters 1, 7 and B. crAss-BACON tandem repeated proteins in these clusters are located in the tail section of the genome. In all clusters except from B, head proteins are those proteins that were identified in the phylogenetic analysis in Figure 2c.

Annotation of the largest contigs in all clusters (Figure 4) indicated that in clusters 2, 5 and 6 the crAss-BACON ORFs are located near head and structural proteins, whereas cluster A crAss-BACON ORFs are located in the transcriptional section of the genome. Considering that crAss-BACON ORFs in clusters 2, 5 and 6 are located among head and structural proteins, they may be phage capsid-decorating binding proteins [2,72]. As only cluster 1, 7 and B ORFs with crAss-BACON tandem repeats are located in the tail section of the genome, these seem to represent examples of the β-hairpin folded repeats that are common in phage tail fibres and spikes [23]. Structural analysis revealed that bacterial BACON domains are linkers that anchor carbohydrate-binding domains to the *Bacteroides* cell surface [12]. Thus, we hypothesised that the tail-associated crAss-BACON tandem repeats may have been recruited by crAss-like phages to bind to the *Bacteroides* cell surface, and that the evolutionary expansion of the tandem repeats that are specific to tail-associated crAss-BACON ORFs may have enhanced the binding capability of the phage tails.

### 3.4. Recurrent Evolution of BACON Domain Tandem Repeats in Phages

As shown above, crAss-BACON tandem repeats are associated with tail proteins in taxonomically distinct phages. Next, we focused on how these repeats evolved. In phage clusters where crAss-BACON tandem repeats were found (1, 7 and B), the genomic regions that contained crAss-BACON ORFs had high sequence similarity, whereas the rest of the phage genomes did not (Figure 4). This suggests that horizontal transfer of the region containing the crAss-BACON ORF has occurred between these phage lineages and their hosts within the crAss-BACON ORFs, as has been previously observed for phage immunoglobulin domains [14]. Vertical transfer of the crAss-BACON ORFs cannot fully explain the similarity of crAss-BACON domains between these phages. The genomes of clusters 1, 7 and B show very little similarity, as shown in Figure 4 and as exemplified by the low conservation or even absence of genes such as the terminase (Figure 2b). 

**Figure 5:**
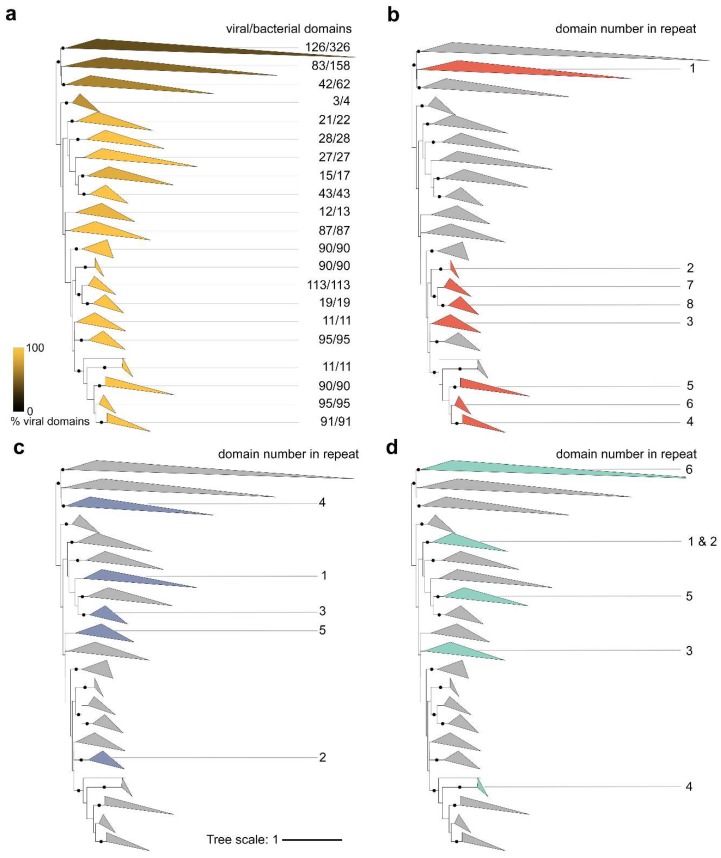
Domain phylogeny of crAss-BACONs in bacteria and phages reveals that the cluster 1 crAss-BACON tandem repeat resulted from multiple duplication events, whereas those in clusters 7 and B resulted from both horizontal transfer and duplications. Displayed is an unrooted approximate maximum likelihood tree [51] of 1205 crAss-BACONs and 304 BACON domains from *Bacteroidetes* species (those that were used to produce PF13004). Dots on branches denote bootstrap support >90. The four versions of the tree depict the fraction of crAss-BACONs out of the total number of domains in each clade (**a**), and the locations of domains from the crAss-BACON tandem repeats in cluster 1 (**b**), 7 (**c**) and B (**d**). Shading and numbers in (**a**) denote the fraction of viral domains in each clade. Numbered clades in (**b**–**d**) represent the positions of domains in the crAss-BACON tandem repeat as counted from the N-terminus. For uncollapsed tree, see Figure S4, Supplementary Materials.

To investigate BACON domain evolution in phages and bacteria, we created a phylogeny of 1509 BACON domains (Figure 5a). This included all 1205 crAss-BACON domains found here and the 304 bacterial BACON domains from which PF13004 was made. Figure 5b–d depicts the crAss-BACON phylogenetic tree with colours indicating clades that contain domains from the crAss-BACON tandem repeats found in clusters 1, 5 and B, respectively. In cluster 1, domains that occupy a specific position in the crAss-BACON tandem repeat (e.g., first domain from the N-terminus, referred to with numbers in Figure 5b–d) each formed a distinct clade (Figure 5b). As domains that occupy a specific position in the cluster 1 crAss-BACON tandem repeat form distinct clades, with no overlap between them, the domain order of the arrays is conserved. This illustrates the evolutionary stability of the cluster 1 crAss-BACON tandem repeat. The clade that contains the first domain in the cluster 1 array (counted from the N-terminus) also contained a number of bacterial domains, whereas no bacterial domains are found in the clades of the seven other domains (Figure 5a). This reveals that this crAss-BACON tandem array resulted from domain duplications within the cluster 1 phage lineage. As the first domain is more closely related to bacterial domains than the other domains in the tandem array, it may be the ancestral domain from which the array expanded. The tree contains two branches that contain cluster 1 crAss-BACON domains that occupy positions 1, 7 and 8 in the array, and those at positions 3, 4, 5 and 6. This means that, similar to domain tandem repeat expansions in cellular organisms [26], cluster 1 crAss-BACON expansion occurred by internal duplications, rather than exclusively at the N- and C-termini (Figure 5b). However, unlike cellular organisms [26], cluster 1 crAss-BACON expansion involved several single domain duplications instead of simultaneous duplication of multiple domains (Figure S3, Supplementary Materials).

The crAss-BACONs in the cluster 7 and B tandem arrays showed a greater spread in the tree (Figure 5c,d). Like cluster 1, these two clusters each contained one crAss-BACON that is closely related to bacterial domains (domain 4 in cluster 7 and domain 6 in cluster B). A further domain from each was closely related to cluster 1 crAss-BACONs (2/7 and 4/B), which seems to be the result of horizontal domain transfer. Each cluster also contained one domain in an isolated branch (5/7 and 3/B) and a number of domains that formed a separate branch (1/7, 3/7, 1/B, 2/B, 5/B). These latter domains resulted from duplications, whereas those that were closely related to bacterial domains may be the ancestral domains. The internal divergence of the domains in these tandem repeats as well as the frequent horizontal transfer of BACON domains between phages and bacteria make it difficult to fully ascertain their evolutionary history.

Our domain phylogeny uncovered an extensive evolutionary history of domain duplications that is characteristic to cluster 1 crAss-BACON ORFs. Identification of additional sequences from clusters 7 and B may further clarify the evolution of crAss-BACON tandem repeats in these clusters. As the cluster 1 crAss-BACON ORF is likely a tail protein, elucidation of its function may provide insight into the host interactions of the most widespread gut phage. The first crAss-like phage that was recently isolated (φcrAss001 [63]) belongs to the heterogeneous candidate genus VI and not to the *Alphacrassvirinae* candidate subfamily that contains the longest and most conserved crAss-BACON tandem array (cluster 1, see Figure 2 and Figure S1). Interestingly, our distant homology searches did not identify any crAss-BACONs in the φcrAss001 genome. Determination of the function of the tail-associated crAss-BACON ORF thus awaits the isolation of crAss-like phages from clusters 1, 7 or B.

## 4. Conclusions

We investigated the evolution of BACON domain tandem repeats in crAssphage and other gut bacteriophages. We demonstrated that crAss-like phage BACON domains (crAss-BACONs) are widespread in the crAss-like phage family, and we identified crAss-BACONs in two novel gut phage lineages. We further found that crAss-BACON tandem repeats are associated with tail fibres in three gut phage lineages. In two lineages (clusters 7 and B), these repeats are the result of horizontal transfer and tandem duplication. The third lineage with tail-associated crAss-BACON tandem repeats includes the prototypical crAssphage (cluster 1, candidate subfamily *Alphacrassvirinae*). In this lineage, we showed how a stable tandem repeat of eight crAss-BACONs has resulted from multiple single domain duplication events. The presented results show that focus on uncharacterised proteins can provide insight into the enormous biodiversity and evolutionary dynamics of the viral world.

## Supplementary Materials

The following are available online at https://www.mdpi.com/1999-4915/11/12/1085/s1, Figure S1: Silhouette plot of the seven crAss-BACON-containing contig clusters shows that cluster 6 is weakly clustered due to heterogeneity, Figure S2: Cluster 1 crAss-BACON ORFs are conserved, except for their C-termini, Figure S3: A similarity matrix of crAssphage BACON tandem repeats shows that the tandem array expansion occurred through single domain duplication events, Figure S4: The same approximate likelihood tree as depicted in Figure 5a, but without collapsed branches, Table S1: crAss-BACON containing contigs, Table S2: crAss-BACON domains, Table S3: Annotation of cluster A contig, Table S4: Annotation of cluster B contig.
