# Supplementary material for: Evolution of BACON Domain Tandem Repeats in crAssphage and Novel Gut Bacteriophage Lineages
Source: Viruses. 2019 Nov 21;11(12):1085. doi: 10.3390/v11121085 (PMC6949934; doi:10.3390/v11121085)
Supplement: Supplementary file 1 [file viruses-11-01085-s001.zip › viruses-643817-final check-supplementary/Supplementary Figures.pdf]

viruses

MDPI

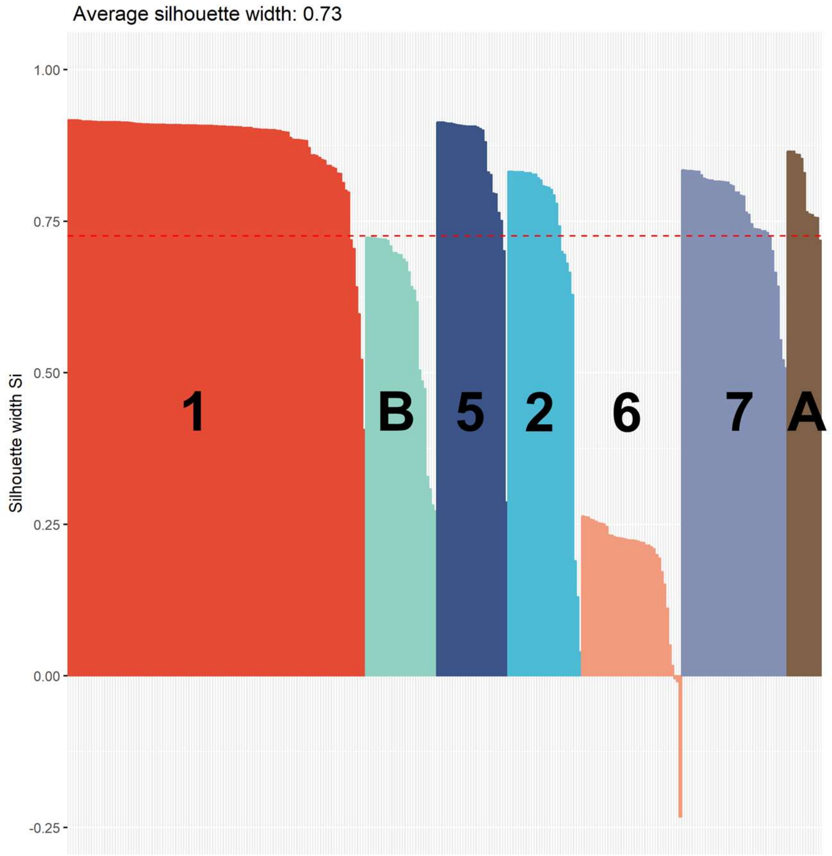

Supplementary Figure 1: Silhouette plot [1] of the seven crAss-BACON-containing contig clusters shows that cluster 6 is weakly clustered due to heterogeneity (see also Figure 2a). Each bar represents one contig, while silhouette width (y-axis) denotes the similarity of that contig to other contigs in the same cluster. Clustering quality decreases with decreasing silhouette width.

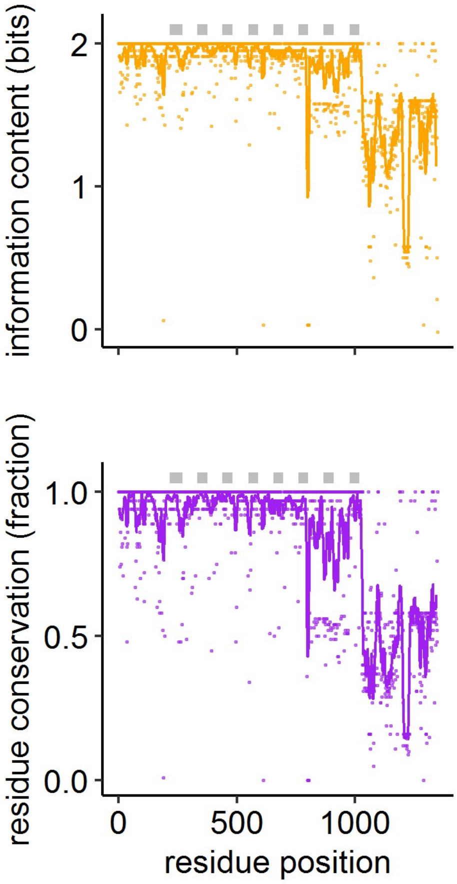

Supplementary Figure 2: Cluster 1 crAss-BACON ORFs are conserved, except for their C-termini. A Clustal Omega [44] multiple sequence alignment was made of all BACON ORFs from cluster 1 that contained eight domains. The top graph represents the likelihood that the position contains a residue, the bottom graph represents the fraction of sequences which has a similar residue. Points are the individual positions, lines are a moving average with a period of 10. Grey blocks at the tops of the graphs are the positions of crAss-BACONs.

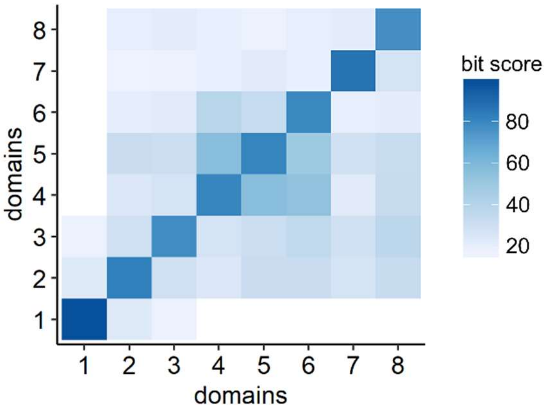

Supplementary Figure 3: A similarity matrix of crAssphage BACON tandem repeats shows that the tandem array expansion occurred through single domain duplication events. Bit scores are the result of a BLASTp all versus all search with crAssphage BACONs. The domain on the x-axis is the query, the domain on the y-axis the hit. Duplication of multiple protein domains at once would result in a checkerboard pattern [26]. Since no such pattern is evident, we conclude that the duplication events involved single domains. Also evident is that the first domain is divergent from the other seven, and that domains 4-6 duplicated internally.

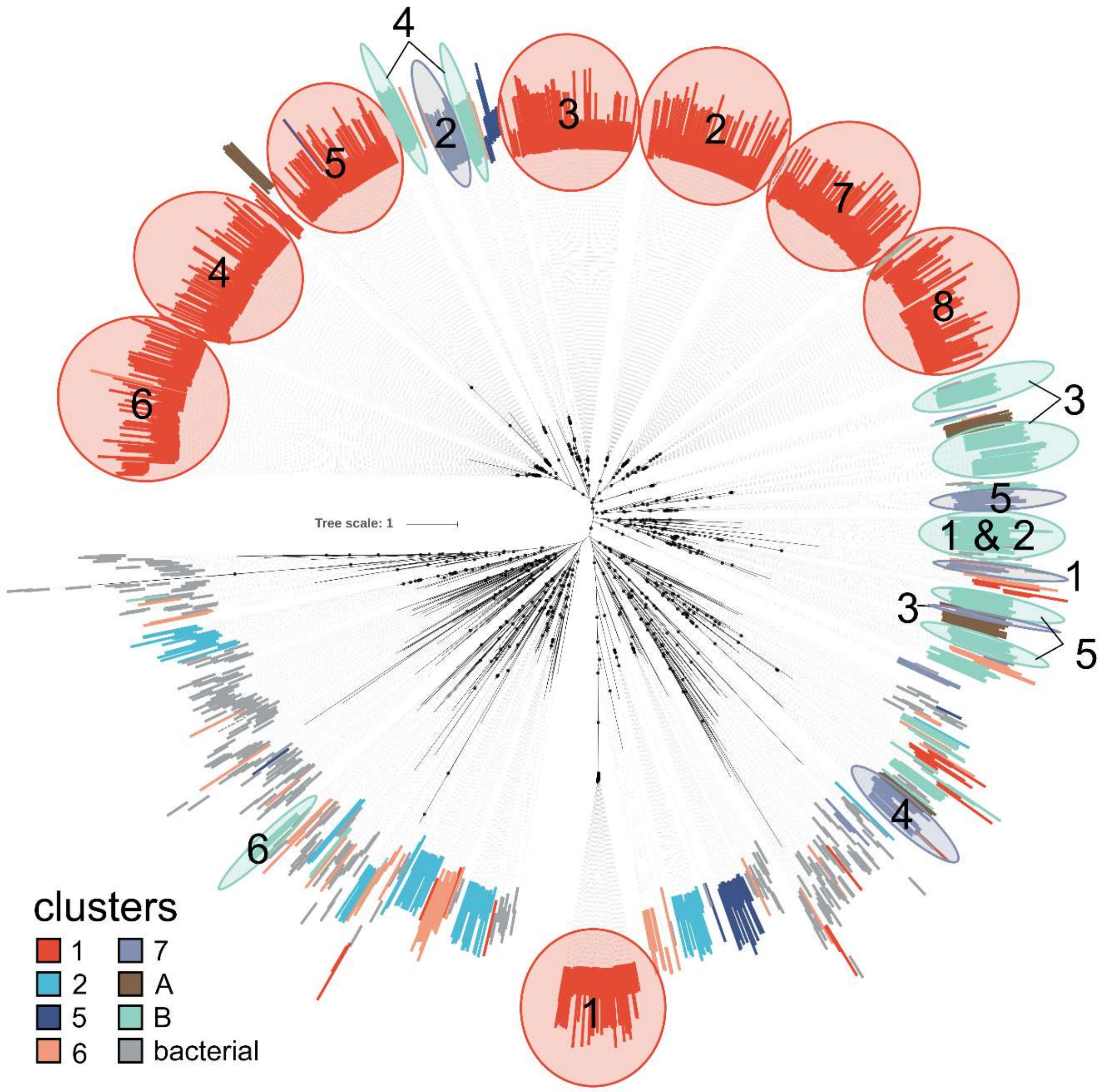

Supplementary Figure 4: The same approximate likelihood tree as depicted in Figure 5a, but without collapsed branches.

1. Rousseeuw, P.J. Silhouettes: A graphical aid to the interpretation and validation of cluster analysis. *J. Comput. Appl. Math.* **1987**, *20*, 53–65.
